# EV20-Sap, a novel anti-HER-3 antibody-drug conjugate, displays promising antitumor activity in melanoma

**DOI:** 10.18632/oncotarget.20728

**Published:** 2017-09-08

**Authors:** Emily Capone, Francesco Giansanti, Sara Ponziani, Alessia Lamolinara, Manuela Iezzi, Annamaria Cimini, Francesco Angelucci, Rossana La Sorda, Vincenzo De Laurenzi, Pier Giorgio Natali, Rodolfo Ippoliti, Stefano Iacobelli, Gianluca Sala

**Affiliations:** ^1^ Department of Medical, Oral and Biotechnological Sciences, University of Chieti-Pescara, Chieti, Italy; ^2^ Department of Life, Health and Environmental Sciences, University of L'Aquila, Coppito (AQ) Italy; ^3^ MediaPharma s.r.l., Via della Colonnetta, Chieti, Italy; ^4^ Department of Medicine and Aging Science, Center of Excellence on Aging and Translational Medicine (CeSi-Met), G. D’Annunzio University, Chieti-Pescara, Italy; ^5^ Sbarro Institute for Cancer Research and Molecular Medicine and Center for Biotechnology Temple University, Philadelphia, USA; ^6^ National Institute for Nuclear Physics (INFN), Gran Sasso National Laboratory (LNGS), Assergi, Italy

**Keywords:** HER-3, antibody-drug conjugate, melanoma, saporin, target therapy

## Abstract

Melanoma is the most biologically aggressive skin cancer of well established constitutive and induced resistance to pharmacological treatment. Despite the recent progresses in immunotherapies, many advanced metastatic melanoma patients still face a significant mortality risk. The aggressive nature of this disease sustains an urgent need for more successful, effective drugs. HER-3 - one of the four member of the tyrosin kinase epidermal growth factor receptors (EGFRs) family- is frequently overexpressed in solid tumors, including melanoma. Moreover, up-regulation of HER-3 and its ligand NRGβ-1 are associated with poor prognosis, thus suggesting this receptor as a suitable target for cancer therapy. Several monoclonal antibodies targeting HER-3 are currently available, but preliminary results from clinical testing of these agents reveal a modest efficacy. Thus, a substantial improvement over this immunotherapeutic approach could be offered by an anti-HER-3 based Antibody-Drug Conjugate (ADC). In the present paper, we describe the generation of an ADC obtained by coupling the HER-3 targeting antibody EV20 linked to the plant toxin Saporin (Sap). *In vitro*, this ADC displays a powerful, specific and target-dependent cytotoxic activity which correlates with the degree of expression and internalization of HER-3 on tumor cells. Furthermore, in a murine melanoma model, EV20-Sap treatment leads to a significant reduction of the number of pulmonary metastasis.

## INTRODUCTION

Melanoma is the most deadly skin cancer and its incidence is significantly increased over the past two decades [1]. Despite the revolutionary advancements in FDA-approved immunotherapy for melanoma, including three checkpoint inhibitors (ipilimumab, pembrolizumab, nivolumab), disseminated metastatic melanoma still lacks effective treatment and recurrence of the disease frequently occurs. Therefore, there is a compelling need to develop new therapeutic agents for the management of metastatic disease in a larger number of patients [2]. HER-3 belongs to the epidermal growth factor (ErbB) receptor tyrosine kinase family which includes four members: HER1 (ErbB1), HER2 (ErbB2), HER3 (HER-3) and HER4 (ErbB4) [3]. HER-3 is overexpressed in a variety of human cancers, including melanoma [4–6] and concurrent expression of this receptor together with its major ligand, NRGβ-1, has been associated with a poor prognosis in several malignancies [7, 8].

Importantly, HER-3 has been found to be upregulated in response to different cancer therapeutics, and it is now considered as one of the major players in promoting activation of escape pathways from anti-cancer treatments [9, 10]. Therefore, targeting this receptor may represent an efficient strategy to enhance therapy efficacy in several cancers, including metastatic melanoma. In line with this, several monoclonal antibodies targeting HER-3 have been developed by major pharmaceutical companies, however, results so far obtained have revealed a modest clinical efficacy of this kind of therapy [11]. Recently, we have developed a humanized monoclonal antibody targeting HER-3, named EV20 [10, 12–14]. This antibody inhibits both ligand-dependent and -independent HER-3 activation, thus interrupting downstream signaling. When used as a single agent, EV20 is able to hamper growth of several tumor xenografts, including those originating from melanoma cells. More importantly, the antibody is rapidly and efficiently internalized by HER-3 expressing tumor cells. This latter property makes EV20 a good candidate for the generation of Antibody-Drug Conjugates (ADCs). Here, we describe EV20-Sap, a novel ADC obtained by coupling EV20 to the ribosome inactivating protein Saporin via chemical crosslinking [15, 16]. In HER-3 expressing cancer cell lines, EV20-Sap demonstrated a powerful, specific and target-dependent killing activity. High expression levels of HER-3 in tumor cells correlated with efficient internalization, efficacy and cytotoxic effects *in vitro*. Furthermore, in an *in vivo* metastatcic model of melanoma, EV20-Sap significantly reduced the number and size of lung metastatic lesions.

## RESULTS

### HER-3 expression in human metastatic melanoma

**Figure 1:**
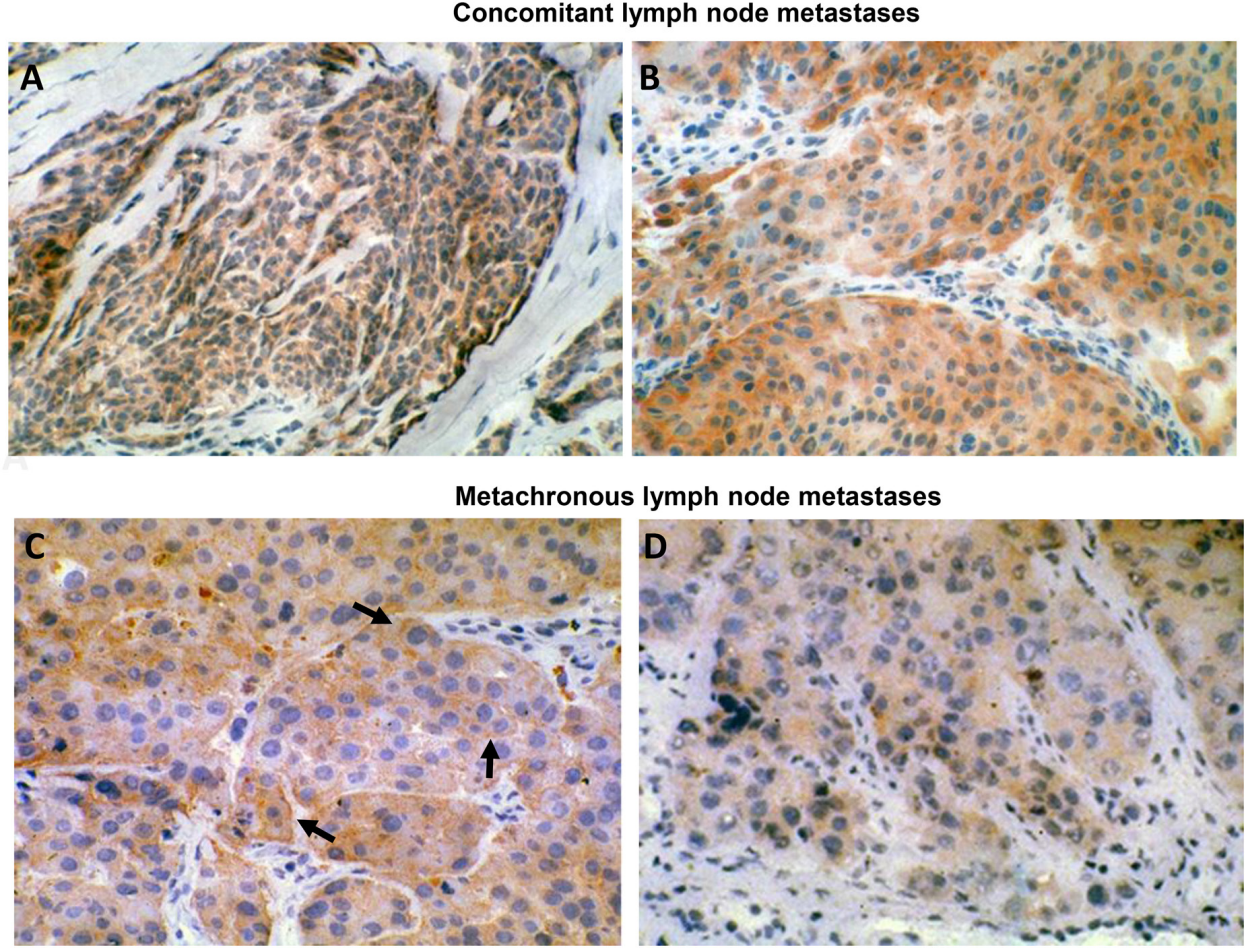
HER-3 expression in human metastatic melanoma **(A)** (160X original magnification) and **(B)** (250X original magnification): concomitant lymph node metastases. **(C)** (250X original magnification) and **(D)** (250X original magnification): metachronous lymph node metastases. Tissues were stained for HER-3 expression using MP-RM-1 antibody. Black arrows indicate MP-RM-1 membrane reactivity.

Among the relevant biological parameters which are mandatory for establishing the potential clinical benefit of an ADC, two of them are of paramount importance, i.e. frequency of the expression of the target molecule in metastatic foci and extent of homogeneity in single lesions. In this context, we submitted our anti-HER-3 antibody to a stringent immune pathological evaluation. Particularly, we assessed HER-3 expression in multiple lymph node metastases, synchronous and metachronous, collected from the same patients as well as in non-lymph node lesions. Indeed, the data reported in Table 1 shows that HER-3, though at different levels, was expressed homogeneously in a significant percentage (average, 74.6%) of the metastases tested, independently of their body location. The expression was almost invariably stable during the course of the disease. Examples of IHC staining of melanoma metastases showing membrane HER-3 positivity are illustrated in Figure 1.

**Table 1:** HER-3 expression in human melanoma metastases

Metastatic site	No. Positive/Total	Score^*^
**Lymph node (synchronous)**	27/38 (71%)	17H; 7T; 3(1+); 11 Neg
**Lymph node (metachronous)**	13/17 (76%)	4H; 8T; 1+; 4 Neg
**Other**	10/13 (77%)	4H; 4T 2(1+); 3 Neg
(Subcutaneous, ovary, lung, adrenal, brain, muscle)		

^*^ Lesions were scored as “negative” when no cellular or interstitial component was stained on a wide range of primary antibody concentrations (5-20 μg/ml) or “Positive” which were in turn classified as “trace (T)” when an homogenous weak stain was detected, or “1+” when an homogenous well defined stain outlined the tumor cells population, “heterogeneous(H)” when tumor cells within a lesion displayed either trace, or 1+ stain, or negative scattered areas which did not exceeded 10% of the whole available sample.

All together it is reasonable to state that an ADC targeting HER-3 may work at its best in metastatic melanoma and may warrant an exploratory clinical assessment.

### EV20-Sap possesses potent and specific cell killing activity *in vitro* and correlates with HER-3 expression in melanoma cell lines

**Figure 2:**
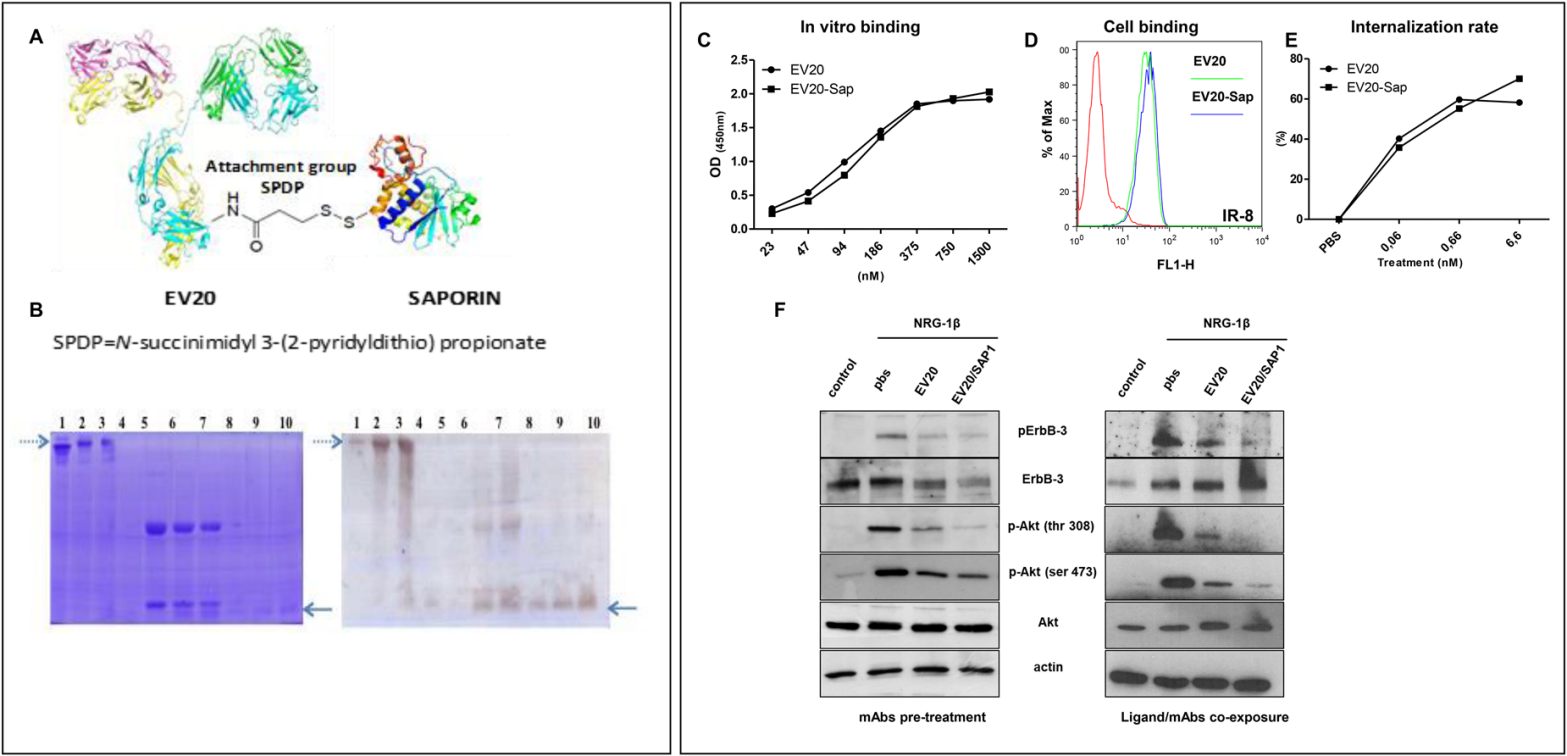
EV20-Sap generation and characterization **(A)** Schematic representation of EV20-Sap. **(B)** Gel electrophoretic analysis of the EV20-Sap product. EV20-Sap (left panel, lanes 2 and 3, dotted arrow) is to be compared with the free antibody (left panel, lane 1). Under reducing conditions (left panel, lanes 6-7), it is possible to observe the release of the toxin moiety that is absent in the free antibody (left panel, lane 5). Saporin position in gel can be seen in the standards of the toxin present on lanes 8-10 (see full arrow). Western blotting using anti-Saporin antibody reveals the toxin in unreduced EV20-Sap (right panel, lanes 2,3 dotted arrow) and the band corresponding to free Saporin in reduced samples (right panel, lanes 6,7, full arrow). Lanes 4, 8-10: free saporin purified from seeds [250 ng, 500 ng, 1000 ng and 1500 ng]. **(C)** ELISA showing *in vitro* binding affinity of naked and Saporin-conjugated EV20 antibody **(D)** FACS analysis showing cell binding of EV20 and EV20-Sap. **(E)** Internalization assay. Surface HER-3 receptor analysis measured by FACS. Human melanoma cells were exposed to increasing concentrations of EV20 and EV20-Sap (0.001-0.01-0.1-1-10 μg/ml) for 6 hrs to 37°C, harvested and stained with 1 μg/million of cells of EV20 for 30 min on ice, followed by Alexa Fluor 488 Goat Anti-Human secondary antibody for 30 min on ice, **(F)** SK-MEL 24 human melanoma cells were starved for 24 hrs and incubated for 2 hrs in presence or absence of 10 μg/ml of EV20 and EV20-Sap, this step was followed by the stimulation with 10 ng/ml of NRG-1β for 5 min (left panel). A375m cells were starved for 24 hrs and incubated simultaneously (15 minutes) with 1 ng/ml of NRG-1β and 10 μg/ml of indicated mAbs (right panel). At the end of the incubation periods, cells were lysed and analysed for pHER-3/HER-3 and pAKT/AKT protein levels by Western blotting. The same filter was reprobed with anti-actin for a loading control.

With the aim of developing a novel agent targeting HER-3 expressing melanoma cells, we have conjugated the anti-HER-3 antibody EV20 with the plant toxin Saporin, obtaining EV20-Sap (Figure 2A-2B). EV20-Sap stability test in complete DMEM and RPMI medium was performed at 37°C for 24 hrs and no Saporin release was observed (data not shown). As shown in Figure 2C-2D, EV20-Sap was able to bind its target with the same efficiency of the naked antibody, as measured by *in vitro* (ELISA) and live cells (FACS) binding. Moreover, conjugation of EV20 did not interfere with the ability of the antibody to promote HER-3 internalization, evaluated by measuring the percentage of receptor loss on cell surface (Figure 2E). Similarly, in cells exposed to the antibodies for 2 hrs before NRG-1β stimulation, EV20-Sap displayed the same ability of the naked antibody in suppressing HER-3 phosphorylation and downstream AKT activation (Figure 2F, left panel). Interestingly, this inhibitory activity was also observed when cells were simultaneously exposed to the ligand and the antibodies (Figure 2F, right panel), confirming an antagonizing effect of naked and conjugated EV20 on NRG-1β driven receptor activation [17].

**Figure 3:**
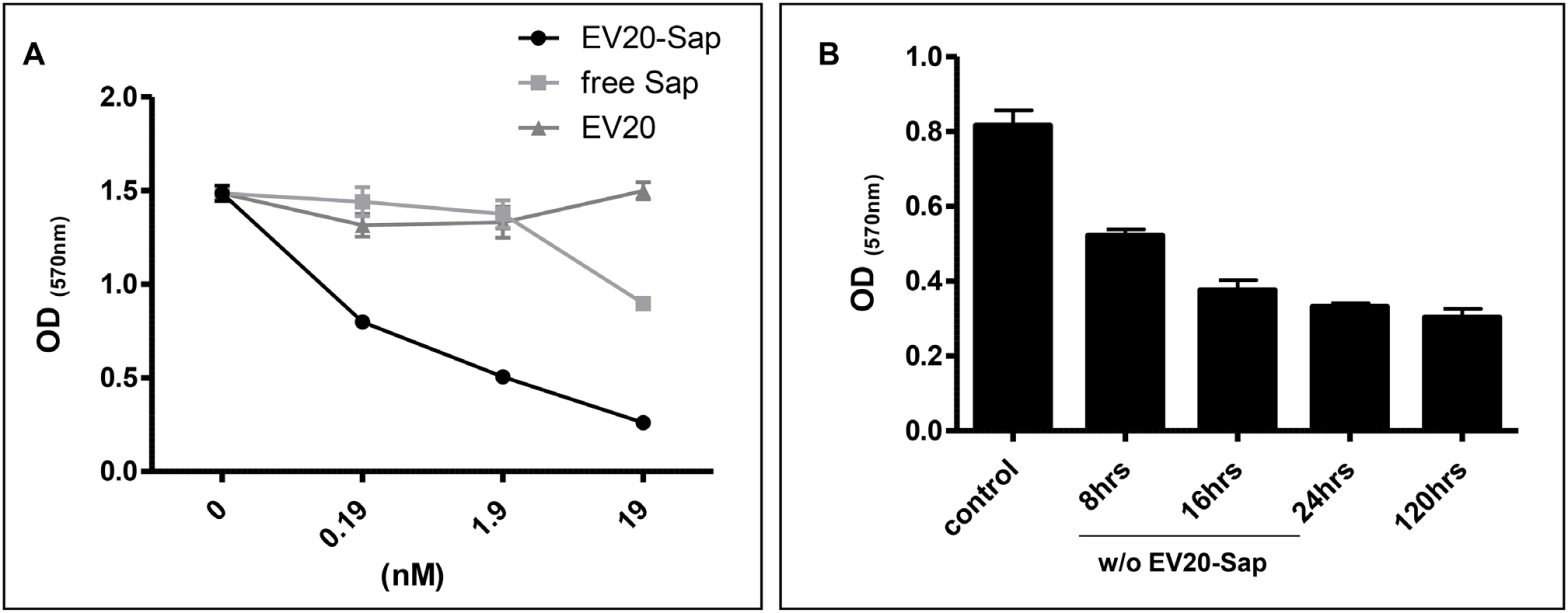
EV20-Sap but not naked EV20 or free Saporin demonstrates cell killing activity in melanoma cells **(A)** SK-MEL 2 cells growing in complete medium were treated with EV20-Sap, EV20 and free Saporin, as indicated. After 5 days cell viability was measured by MTT staining. Mean +/- SD (n=3). **(B)** SK-MEL 24 cells were seeded in 24-wells plate at a density of 5×10^3^ and allowed to attach and propagate overnight before the treatments with EV20-Sap (1.9 nM). The ADC was removed (wash out) at different time points and complete medium replaced until the end of experiment. Cells treated continuously for 120 hrs were used as control. Cell viability was assessed by MTT staining. OD: optical density. Mean +/- SD (n=3).

We moved forward testing the *in vitro* antitumor activity of this novel immunoconjugate in melanoma cells. To this end, we performed a dose response experiment in cells grown in complete medium and treated for 120 hrs with naked mAb, Saporin-conjugated EV20 and free Saporin. Under these conditions, unconjugated EV20 showed no effect in terms of growth inhibition whilst treatment with EV20-Sap resulted in a potent cell killing activity, with only around 10% of cell surviving to the higher doses (19 nM). Importantly, free Saporin in the concentration range explored was scarcely toxic towards the same cell line, thus confirming that the toxic activity of EV20-Sap was mediated by the internalization of the chimeric toxin via HER-3 (Figure 3A). As we have previously demonstrated that EV20 antibody internalization occurs already after 30-60 minutes [12], we investigated the cell killing activity of the ADC after short exposure to test if it would be as efficient as continuous treatment. We therefore performed a time course experiment in which EV20-Sap was removed (wash-out) at different time points or left on cells for the duration of the experiment (120 hrs). We found that EV20-Sap possesses cytotoxic activity already after a short exposure (8 hrs). Notably, 24 hrs exposure produced the same growth inhibition as the continuous incubation (Figure 3B). Taken together these results demonstrate that EV20 antibody could be used to efficiently and rapidly deliver lethal cargoes to HER-3 expressing melanoma cells.

**Figure 4:**
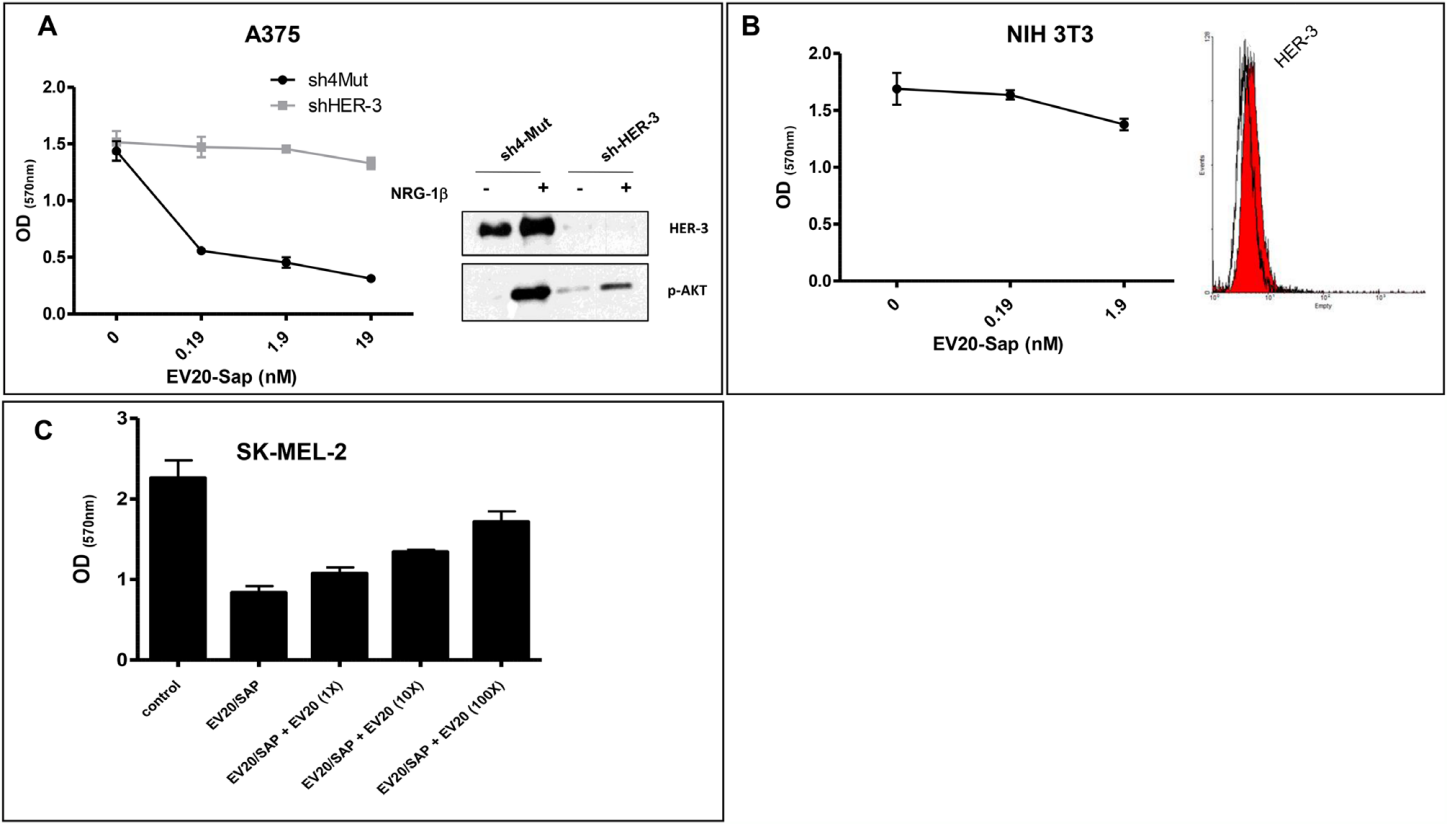
EV20-Sap antitumor activity is target-dependent **(A)** Effect of EV20-Sap treatment was evaluated in HER-3 silenced A375m cells. 4Mut (control) and shHER-3 A375m cells were treated with of EV20-Sap for 5 days and proliferation assay performed by MTT staining. Mean +/- SD (n=3). Efficiency of HER-3 knock-down was measured by western blotting (right panel). Ligand induced AKT activation was evaluated to confirm HER-3 knockdown. **(B)** Treatment of murine NIH-3T3 cells with EV20-Sap for 5 days. Surface expression of HER-3 was evaluated by FACS, right panel. **(C)** SK-MEL 2 cells were treated with EV20-Sap (1.9 nM) in the presence or increasing molar excess of naked EV20. After 5 days, cells viability was determined by MTT staining. OD: optical density. Mean +/- SD (n=3).

In order to demonstrate the selectivity and specificity of EV20-Sap cell killing activity we used A375m cells stably knocked down for HER-3 expression (Figure 4A). We found that EV20-Sap cytotoxic activity was completely lost in HER-3 silenced cells but not in control (4mut) cells, thus indicating that the ADC required the expression of the target for delivering its cytotoxic compound into the cancer cells. In keeping with this observation, no significant cytotoxic effect was seen in NIH3T3 fibroblasts, which do not express HER-3 (Figure 4B). Next, we wondered whether the loss of EV20-Sap activity observed in murine cells was due to lack of HER-3 expression or to EV20 inhability to recognize murine HER-3. To this aim, we took advantage of HER-3+ BALB-neu T mouse-derived mammary lobular carcinoma cells (TUBO) and commercial C-17 anti-HER-3 antibody, which recognizes both mouse and human HER-3 receptors, as evaluated by flow cytometric analysis and western blotting (Supplementary Figure 1A-1B). By contrast, EV20 reactivity on cell surface, as evaluated by FACS analysis, was observed in human (IR-8) but not in murine (TUBO) cells, thus indicating that this antibody does not bind the murine form of HER-3 receptor. In agreement with this observation, no cell killing activity was detected in TUBO cells exposed to increasing doses of EV20-Sap (Supplementary Figure 1D). Finally, EV20-Sap antitumor activity was almost fully competed by a 100-fold molar excess of naked EV20 (Figure 4D), clearly proving that Saporin internalization into cancer cells is EV20-dependent.

**Figure 5:**
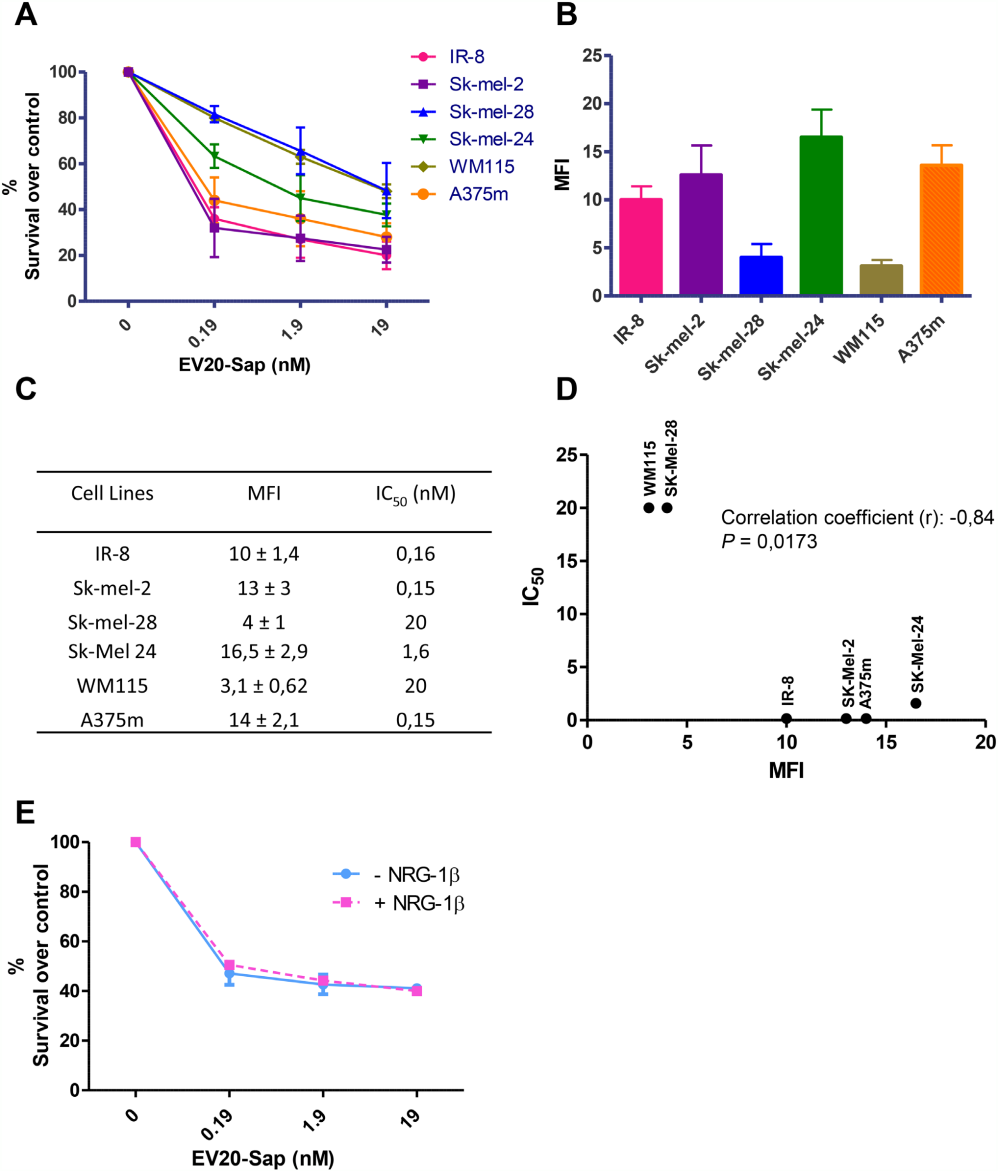
EV20-Sap IC50 correlates with HER-3 surface expression **(A)** EV20-Sap cell killing activity was evaluated in a panel of melanoma cells. Cells were treated with increasing doses of EV20-Sap for 120 hrs, proliferation was assessed by MTT assay and results are shown as % of control (PBS treated cells). Mean +/- SD (n=3). **(B)** Surface expression of HER-3 receptor evaluated by FACS analysis in melanoma cells. Mean +/- SD (n=3). **(C)** MFI and IC_50_ values are reported for all cell lines tested. **(D)** EV20-Sap IC_50_ and surface HER-3 expression was plotted and correlation coefficient (r) calculated by GraphPad Prism 5 software (*p*<0.01). **(E)** EV20-Sap cell killing activity (120 hrs treatment) was evaluated in A375m cells in presence or not of HER-3 ligand NRG-1β (10 ng/ml). Cell viability was assessed by MTT assay and results are shown as % of control (PBS treated cells). Mean +/- SD (n=3).

We then evaluated the cell killing activity of EV20-Sap in a panel of human melanoma cell lines with different levels of HER-3 expression. Interestingly, we observed that EV20-Sap IC_50_ (which ranged from 0.15 to 20 nM) significantly correlates with HER-3 surface expression (correlation coefficient (r) = -0.84; *p*=0.017) (Figure 5A-5D). Finally, we asked whether the activity of our ADC would be affected by the presence of receptor ligand. To this aim, A375m melanoma cells were treated with increasing doses of EV20-Sap in the presence or not of NRG-1β (10 ng/ml). EV20-Sap cell killing activity resulted to be unchanged by the presence of HER-3 ligand (Figure 5E).

Overall, these data firmly indicate that the cytotoxic activity of EV20-Sap is the result of the HER-3 mediated internalization of the immunotoxin in the target cells.

### EV20-Sap reduces lung metastatic lesions of melanoma cells

**Figure 6:**
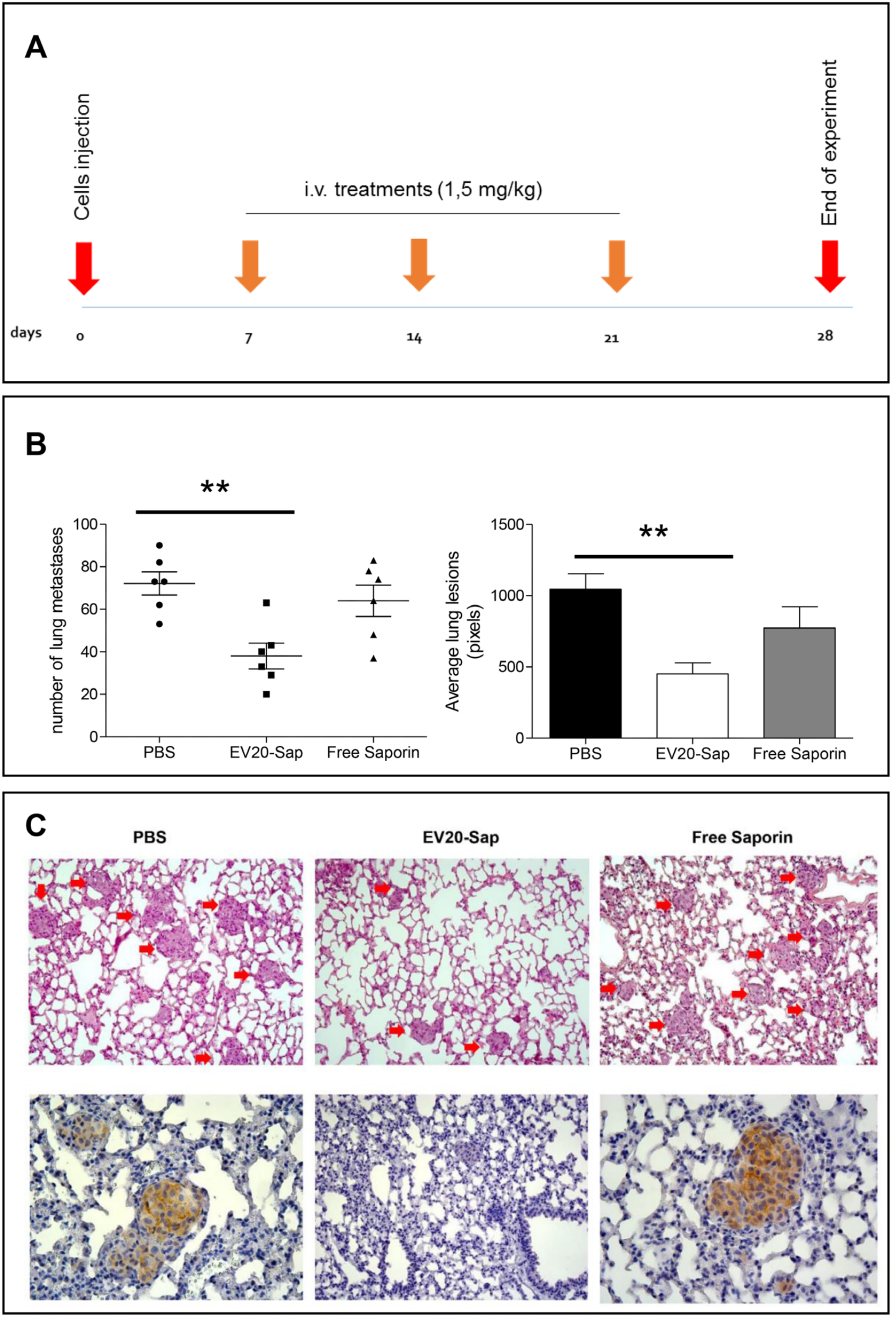
EV20-Sap shows a potent *in vivo* antitumor activity in a model of melanoma experimental metastasis **(A)** Schematic representation of experimental lung metastasis assay. **(B)** Number (left) and size (right) of lung metastatic lesions evaluated at the end of the experiment in control (n=6), EV20-Sap (n=6) and free Saporin (n=6) treated animals. Error bars represent SD. *p* values were determined by Student's *t* test. ^**^p<0.01. **(C)** Representative images of haematoxylin and eosin (H&E) of lung sections of control and treated groups (upper panel) and corresponding immunohistochemical staining of HER-3 on metastatic lesions (lower panel).

In order to translate these findings into potential pre-clinical relevance we tested the therapeutic efficacy of EV20-Sap *in vivo* on a mouse model of experimental lung metastasis. To this aim, we used invasive SK-MEL 24 melanoma cells which demonstrated the ability to form metastatic lesions in the lungs in 100% of animals 14 days after injection of cells (data not shown). The amount of ADC used in this *in vivo* setting was established after a preliminary dose-escalation study where 1.5 mg/kg resulted as the maximum tolerated dose (MTD). Mice were intravenously injected with 0.5×10^6^ SK-MEL 24 cells and treated with PBS, EV20-Sap (1.5 mg/kg) or free Saporin (1.5 mg/kg), three times weekly, starting one week (day 7) after cell injection and sacrificed at day 28 (Figure 6A). As shown in Figure 6B, treatment of animals with EV20-Sap, but not with free Saporin, significantly reduced both the number and the size of pulmonary metastases, as compared to animals receiving the vehicle (PBS: 72 ± 13; EV20-Sap: 38 ± 6; free Sap: 64 ± 18. PBS vs EV20-Sap; *p* = 0.01). As expected, no toxicity was observed in EV20-Sap treated animals in terms of body weight loss (data not shown). Finally, immunostaining of lung sections confirmed high and membranous expression of HER-3 on metastatic lesions (Figure 6C).

## DISCUSSION

Over the past 30 years, we have witnessed a remarkable and significant advancement in the efficacy of therapies in several types of cancer, including melanoma. This was essentially due to the development of drugs designed to block specific pathways known to support tumour progression. However, despite these partial successes, still a large number of patients with an advanced stage, die as a consequence of metastatic drug resistant disease. This is particular true for melanoma where current therapies show only limited efficacy in patients with late-stage metastatic disease.

Experimental, preclinical and clinical data indicate that HER-3 plays an important role in the progression of melanoma [18]. Several monoclonal antibodies targeting HER-3 are now being developed by major pharmaceutical companies. Among them, six naked antibodies are in clinical development (phase II/phase III): U1287 from Daiichi-Sankyo, MM-121 from Merrimack Pharmaceuticals, LJM716 from Novartis, KTN3379 from Kolltan Pharmaceuticals, GE-huMab-HER3 from Roche and AV-203 from Aveo Pharmaceutical. The use of naked antibodies directed against HER3 is certainly an interesting approach to treat patients with HER3-positive cancers, however preliminary results from the clinical trials reveal only a modest effect of this agents [11, 19–21].

Thus, a substantial improvement over this immunotherapeutic approach could be offered by an HER-3-based Antibody Drug Conjugate (ADC). ADCs are constituted by a recombinant antibody covalently bound by a synthetic linker to a given cytotoxic agent, either drug or toxin. The main objective of the ADCs is to confer higher tumor selectivity to cytotoxic agents that are too toxic to be used on their own and cell killing power to monoclonal antibodies that are tumor-specific but not sufficiently cytotoxic [22–24]. To date only three ADCs, gemtuzumab ozogamicin (anti-CD33 antibody conjugated to calicheamicin), brentuximab vedotin (anti-CD30 conjugated to auristatin E), T-DM1 (which consists of the cancer-killing agent emtansine or DM1 linked to the HER2-binding antibody, trastuzumab), have been approved by Food and Drugs Administration [25, 26].

In this study, we have developed a novel ADC targeting HER-3 by the conjugation of EV20 to the toxin Saporin. Saporin belongs to the ribosome inactivating protein family (Type I RIPs) [27, 28] widely used to construct immunotoxins or chimeric toxins (linking the toxic moiety to a carrier other than Abs) [29]. Saporin becomes cytotoxic upon internalization, thus we proved that EV20 antibody can be efficiently conjugated to carry Saporin to target cancer cells overexpressing this receptor. Using a chemical crosslinking procedure, we obtained a homogeneous product containing one Saporin molecule linked to one antibody molecule (EV20-Sap). This conjugate maintained the biological activity of the naked antibody (it binds to melanoma cells with the same affinity as free EV20) and of the toxin (it intoxicate cells upon internalization with IC_50_ values typical of Saporin-based immunotoxins, i.e. in the range of 0.15 to 20 nM). The data reported in Table 1 demonstrated that HER-3, though at different levels, is expressed homogeneously in a significant percentage (76%) of metastatic melanoma lesions tested, independently of their body location and sequence of appearance. The expression was almost invariably stable during the course of the disease, as judged by the same value of HER-3 staining scoring in synchronous and metachronous metastases. For IHC analysis, we used the murine MP-RM1 which reacts only on frozen sections. On these sections, the staining pattern was predominantly cytoplasmic although in some lesions a clear cell membrane staining was observed (black arrows on Figure 1). This staining pattern on frozen sections is not unique of MP-RM1 but is shared also by other anti HER-3 antibodies, including a rabbit monoclonal (Ab-Cam antibody SP-71) and a polyclonal antibody (Santa Cruz C-17) [18]. Indeed, parallel testing of a representative number of metastatic melanoma lesions with MP-RM1 and the two above mentioned antibodies showed overlapping cytoplasmic staining patterns (data not shown). The only differences were limited to a lower intensity of stain with MP-RM1. Though any explanation for different staining patterns related to the type of tissue processing (paraffin vs cryostat sections) is at this stage hypothetical, the overlapping reactivity suggests that testing tissue samples with paraffin reacting reagents mirrors that of MP-RM1.

EV20-Sap *in vitro* activity on melanoma cells was proportional to HER-3 expression and not affected by the presence of receptor ligand NRG-1β, suggesting the potential benefit of this immune-conjugate in the treatment of most aggressive tumors i.e., those exhibiting a concomitant expression of both HER-3 and its ligand NRG-1β [30]. Moreover, EV20-Sap was effective *in vivo* in reducing the size and the number of pulmonary metastases in a model of metastatic melanoma.

In summary, the results presented in this study confirm that HER-3 might be a potential therapeutic target for metastatic melanoma. Moreover, they demonstrate that HER-3 positive metastatic melanoma can be specifically and effectively targeted by the anti HER-3 antibody EV20 conjugated to the plant toxin Saporin, as in the present study, or potentially to other cytotoxic drugs. The therapeutic efficacy of the EV20-based ADC deserves further preclinical testing in HER-3 positive malignancies besides metastatic melanoma.

## MATERIALS AND METHODS

### Reagents

Antibodies were as follows: phosphorylated Akt (Ser473/Thr308), Akt, phosphorylated ErbB-3 (Tyr1289), ErbB-3, from Cell Signaling Technology (Danvers, MA); C-17 ErbB-3 from Santa Cruz Biotechnology (Santa Cruz, CA); anti-actin was purchased from Sigma-Aldrich Corporation (St Louis, MO). Neuregulin-1β (NRG-1β) was purchased from R&D Systems, Inc (Minneapolis, MN). Recombinant human ECD ErbB-3 was from ACROBiosystems (Bethesda, MD). (3-(4,5-Dimethylthiazole-2-yl)-2,5 diphenyltetrazolium bromide (MTT) was purchased from Sigma-Aldrich Corporation. EV20 antibody was produced as described [12].

### Cell lines

Melanoma (A375m, Sk-mel 2, Sk-mel 24, Sk-mel 28, WM115) and murine fibroblast NIH 3T3 cells were purchased from American Type Culture Collection (Rockville, MD, USA). Her-2/neu positive TUBO cell line has been originally isolated from a carcinoma arising in a BALB-neuT mouse [31] and was cultured in DMEM with 10% FBS. IR-8 melanoma cells were previously described [17]. The cells were grown with media according to manufacturer instructions supplemented with 10% heat-inactivated fetal bovine serum (FBS; Invitrogen), l-glutamine, 100 units/ml penicillin, and 100 μg/ml streptomycin (Sigma-Aldrich Corporation, St. Louis, MO, USA), and incubated at 37 °C in humidified air with 5% CO2. For stable HER-3 silencing, sequence targeting the human HER-3 mRNA (Lee-Hoeflich et al., 2008) was subcloned into pSuper vectors. Control vector pSuper 4Mut contains a four-point mutated sequence unable to target the human HER-3 mRNA. Silencing of HER-3 was obtained by using pSuper retro-based vectors to express stable RNA (shRNA). Cells were infected with pSuper retro–based vectors as described [32].

### Sap-SO6 purification

Saporin was purified from the seeds of Saponaria officinalis as described [33]. The fractions containing Saporin SO6 were analyzed by SDS-PAGE, pooled and their concentration estimated by spectrophotometer reading at 280 nm using an extinction coefficient (ε) equal to 0.7 mg/ml. The Saporin preparation was dialyzed in 20mM Borate buffer pH 9 and stored at -80°C.

### Generation of EV20-Sap

Purified Saporin was chemically conjugated to EV-20 antibody by introducing a disulfide bridge by 2- iminothiolane and N-succimidyl-3-(2-pyridyldithio) propionate (SPDP) (Thermo Scientific) as previously described [34]. Purification of EV20-Sap was done as already described [15]. The concentration of EV20-Sap was determined at 280 nm using an estimated extinction coefficient (ε) equal to 1.3 mg ml-1 (calculated on the basis of EV20-Sap on http://www.expasy.ch/tools/protparam.html). EV20-Sap fractions were stored at -80°C until used.

### ELISA

Ninety-six well-plates (NUNC Maxisorp modules) were coated by incubation overnight at 4°C with human recombinant HER-3 extracellular domain (ECD) (1 μg/ml). After blocking with 1% BSA in PBS + 0,1% Tween-20 for 1 hour at room temperature, increasing concentrations of EV20 or EV20-Sap were added and incubated at room temperature for 1 hour. After several washes with PBS + 0, 1% Tween-20, goat anti-human IgG-HRP solution (Sigma-Aldrich Corporation) was added and incubated at room temperature for 1 hour. After several washes, stabilized chromogen was added to for at least 10 minutes in the dark, then the reaction was stopped with the addition of H2S04 1N and the absorbance was read at 450 nm with an Elisa reader.

### Internalization assays

A375m cells were plated in 12 well-plates and grown in 10% FBS DMEM for 24 hrs. Cells were then incubated with increasing doses of EV20 or EV20-Sap for 6 hrs for dose-dependent assay; alternatively, cells were incubated with 0.1 μg/ml of EV20 or EV20-Sap for the indicated times for time-dependent assay. Flow cytometry assay was performed as previously described [12].

### FACS analysis

Flow cytometry for receptor surface expression analysis was performed according to the standard procedures. Around one million of cells were harvested and labeled with EV20 or EV20-Sap on ice for 30 minutes. Cells were then washed with 2 ml PBS, pulled down, and stained with an Alexa Fluor 488 Goat anti-Human (Molecular Probes, Life Technologies). For commercial anti-HER-3 C-17 sc staining, cells were fixed and permabilized before antibody incubation.

### Cytotoxicity assay

Cell proliferation was assessed by MTT [3-(4, 5-dimethyldiazol-2-yl)-2,5-diphenyl tetrazolium bromide] assay (Sigma-Aldrich), Trypan Blue staining and cell count. For MTT assay, cell lines were seeded into 24-well plates at a density of 5 × 10^3^ cells/well in 500μl of complete medium, cells were treated with drugs at indicated concentration in triplicates and further incubated for 120 hrs. At the end of the incubation period, cells were incubated with 200 μl of MTT solution (medium serum free with 0,5 mg/ml of MTT) for further 2 hrs. After removal of MTT solution, 200 μl of dimethyl sulfoxide (DMSO) was added to the wells for 10 minutes and the absorption value at 570 nm was measured using a multi-plate reader. All experiments were performed in quadruplicate. *p* values were determined by Student's *t* test and considered significant for *p* < 0.05. For competition assays cells were seeded in 24-wells plate at a density of 5×10^3^ cells/well in 10% fetal bovine serum-containing medium and allowed to attach and propagate overnight before the treatments.

### Experimental lung metastasis

NOD SCID gamma (NSG) mice (8-weeks old) were maintained under specific pathogen-free conditions with food and water provided ad libitum. The animal health status was monitored daily. Procedures involving animals and their care were conducted according to the institutional guidelines in compliance with national and international laws and policies. Mice were injected intravenously with SK-MEL-24 melanoma cells (5×10^5^); after one week (7 days) mice received i.v. injection of 1.5 mg/kg EV20-Sap or PBS (control group). At days 28 mice were sacrificed and lungs were processed for metastasis analysis. Lungs were harvested, fixed in 10% neutral buffered formalin and paraffin embedded. To ensure systematic uniform and random sampling, lungs were cut transversally, to the trachea, into 2.0 mm thick parallel slabs with a random position of the first cut in first 2mm of the lung, resulting in 5-8 slabs for lungs. The slabs were then embedded cut surface down and sections were stained with Haematoxylin and Eosin (BioOptica, Milan, Italy) for detecting lung metastasis. H&E lung sections of each mouse were used to quantify the number and area of metastasis per field. Fifteen 10X fields per lung were analysed and the average number is shown in the graphs. Lungs sections were also stained for HER-3, the used antibody was purchased from CellSignalling Technology.

### Immunohistochemistry

#### Human metastic melanoma

Metastatic melanoma tissues (N=68) from different body sites were made available through a collaboration between MediaPharma Srl, Chieti, Italy, and the “Regina Elena” National Cancer Institute, Rome, Italy. Tissue were collected according to the Regina Elena's Ethical Commitee reccomandations (March 1, 2006, Regina Elena Ethical Committee Official Guidelines). The biological material and pathologic diagnosis was provided to MediaPharma coded without anagraphic information and clinical follow-up data. All tissues were collected from patients free of chemotherapy and/or radiation therapy. Samples were rapidly snap-frozen in liquid nitrogen and stored at -80° C. At least 3 non-consecutive 4u thick sections (Leica cryostat microtome) were harvested on a single tissue slide with frozen band reporting the case identification code. Through this procedure, the assessment of the immune histochemical findings could be established at different histopathological level on one single slide, thus allowing a critical assessment of the extent of homogenous expression of the receptor. After air-drying at room temperature, sections were fixed for 10 min in cold acetone and then either immediately used as a substrate in indirect immunoperoxidase reaction or kept at -20°C without loss of immune reactivity for at least 6 months, as monitored with appropriate control stain. Tissue morphology was assessed using the same section substrates stained with toluidine blue (0.1% in PBS).

Frozen tissue sections underwent immunoperoxidase stain by incubation overnight at 4°C with MediaPharma's antibody MP-RM-1 (murine analog of EV20). After removal of the primary antibody and washing twice with cold PBS, sections were stained with the Vectastain ABC kit (Vector Laboratories, Inc., Burlingame, CA) according to the manufacturer's instructions using aminoethylcarbazole as chromogen. Nuclear stain was done using Mayer's hematoxylin. Finally, slides were mounted in aqueous mounting medium and stored at 4° C with no loss of staining for at least 5 months. The assays included appropriate negative (isotype matched primary irrelevant antibody) and positive control (archival tissue section displaying HER-3 expression). Immunohistochemical findings were recorded blindly by two Pathologists.

## SUPPLEMENTARY MATERIALS FIGURES AND TABLES



## Abbreviations

ADC: antibody-drug conjugate
FACS: fluorescence-activated cell sorting
OD: optical density
IHC: immunochemistry
ECD: extra cellular domain
ELISA: enzyme-linked immunosorbent assay
