# Supplementary material for: EV20-Sap, a novel anti-HER-3 antibody-drug conjugate, displays promising antitumor activity in melanoma
Source: Oncotarget. 2017 Sep 8;8(56):95412–24. doi: 10.18632/oncotarget.20728 (PMC5707031; doi:10.18632/oncotarget.20728)
Supplement: Supplementary file 1 [file oncotarget-08-95412-s001.pdf]

EV20-Sap, a novel anti-HER-3 antibody-drug conjugate, displays promising antitumor activity in melanoma

SUPPLEMENTARY MATERIALS

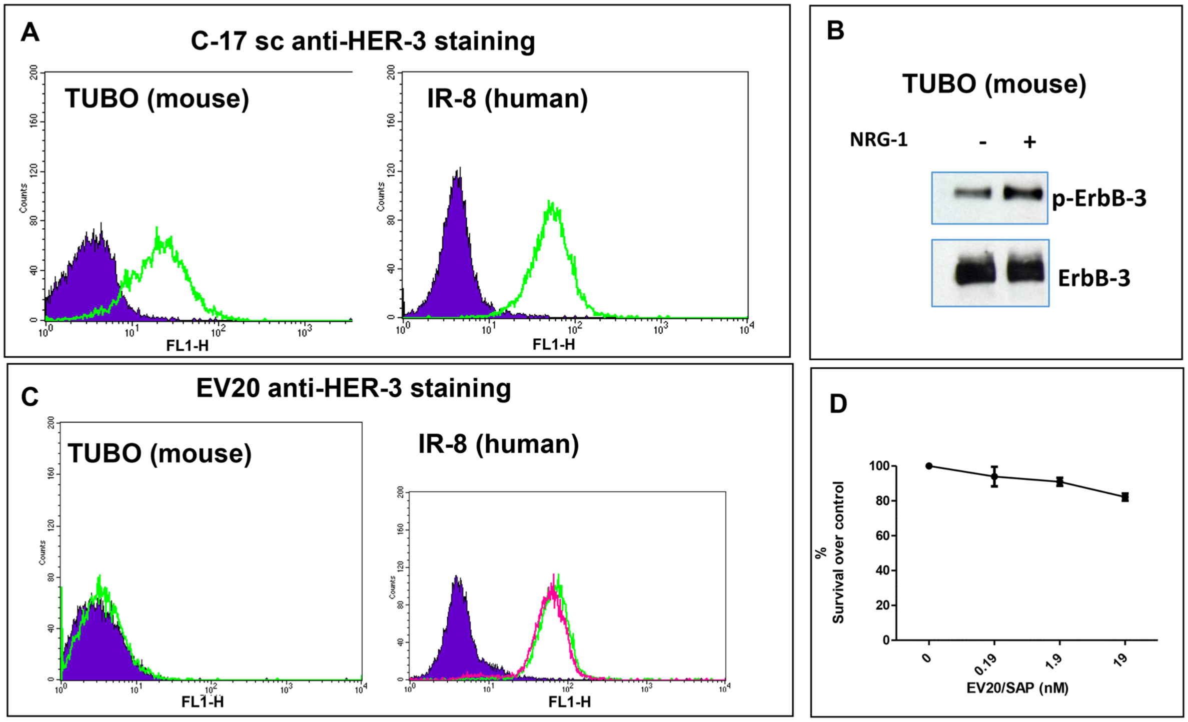

Supplementary Figure 1: EV20-Sap does not recognize murine HER-3 and shows no cell killing activity in HER-3+ BALB-neu T mouse-derived cells (TUBO). **(A)** Murine (left) and human (right) HER-3 expression was analyzed by FACS using the commercial anti-HER-3 antibody C-17 sc followed by Alexa fluor 488 goat anti-rabbit IgG staining. **(B)** Murine HER-3 expression and activation upon ligand stimulation was evaluated by western blotting in murine TUBO cells. **(C)** Murine (left) and human (right) HER-3 expression was analyzed by FACS using the EV20 or EV10 antibody followed by Alexa fluor 488 goat anti-human IgG staining. Green line: EV20 mAb; purple line: EV10 mAb [13]. **(D)** EV20-Sap cytotoxic activity was evaluated in murine TUBO cells after 120hrs of treatment. Cell viability was assessed by MTT assay and results are shown as % of control (PBS treated cells). Mean +/- SD (n=3).
